# Radiomics approach for identifying radiation-induced normal tissue toxicity in the lung

**DOI:** 10.21203/rs.3.rs-3951996/v1

**Published:** 2024-02-23

**Authors:** Olivia GG Drayson, Pierre-Montay Gruel, Charles L. Limoli

**Affiliations:** University of California, Irvine; University of Antwerp; University of California, Irvine

**Keywords:** Radiation-induced lung injury, Radiomics, Extracellular vesicles

## Abstract

Radiomic features were used in efforts to characterize radiation-induced normal tissue injury as well as identify if human embryonic stem cell (hESC) derived Extracellular Vesicle (EV) treatment could resolve certain adverse complications. A cohort of mice (n=12/group) were given whole lung irradiation (3×8Gy), local irradiation to the right lung apex (3×12Gy), or no irradiation. The hESC-derived EVs were systemically administered three times via retro-orbital injection immediately after each irradiation. Cone-Beam Computed Tomography (CBCT) images were acquired at baseline and 2 weeks after the final radiation/EV treatment. Whole lung image segmentation was performed and radiomic features were extracted with wavelet filtering applied. A total of 851 features were extracted per image and recursive feature elimination was used to refine, train and validate a series of random forest classification models. Classification models trained to identify irradiated from unirradiated animals or EV treated from vehicle-injected animals achieved high prediction accuracies (94% and 85%). In addition, radiomic features from the locally irradiated dataset showed significant radiation impact and EV sparing effects that were absent in the unirradiated left lung. Our data demonstrates that radiomics has the potential to characterize radiation-induced lung injury and identify therapeutic efficacy at early timepoints.

## Introduction

Radiomics is an emerging field that involves the extraction and analysis of quantitative data from medical images. The technique was developed to extract more information on tumor tissue that supplements clinical data and can better predict tumor progression. These data include types of features such as intensity, shape, and texture which can provide information about tissue characteristics and the biological behavior of tumors. In conjunction with machine learning, radiomic data can be used to improve diagnosis, prognosis, and treatment planning for cancer patients. It can also help to identify biomarkers that can predict response to therapy and guide personalized treatment. Combined with other “omic” data in what is now called “multiomics”, radiomics is an essential part of modern personalized oncology.

In the field of oncology, radiomics is of particular interest in assessing the efficacy of radiotherapy and chemotherapy treatments. Radiomics can be applied to assist in staging of the disease, identification of genetic features, discrimination of healthy and unhealthy tissue for treatment planning and treatment monitoring through prediction of remission, treatment outcome or survival^1^. While radiomics is a tool that originated from and is predominantly utilized in the field of oncology, it is starting to be applied to other human diseases. Radiation toxicity and radiosensitivity is beginning to be investigated with radiomics^2,3^, as well as diseases and health effects that impact whole organs such as liver fibrosis^4^, impaired pulmonary function^5^, and MRI functional brain activity^6^.

In a previous study, it was demonstrated that retro-orbital injection of stem-cell-derived extra-cellular vesicles into mice was able to improve survival and mitigate lung fibrosis induced by a single dose of X-ray irradiation^7^. Therefore, this study was repeated with two hypofractionated irradiation paradigms: a whole-lung irradiated cohort and a locally irradiated cohort. CBCT imaging was conducted at baseline, 2 weeks after IR and EV injection. Our study shows that, while experimenter-dependent 2D contouring quantification was not able to identify changes in lung density between the different treatment groups, radiomics analysis was able to predict both radiation and EV treatments with significant accuracy. These results highlight the possible use of radiomics in the early detection of radiation-induced injury.

## Results

### Experimenter-dependent 2D lung density measurements fail at identifying radiation-induced lung injury

**Figure 1:**
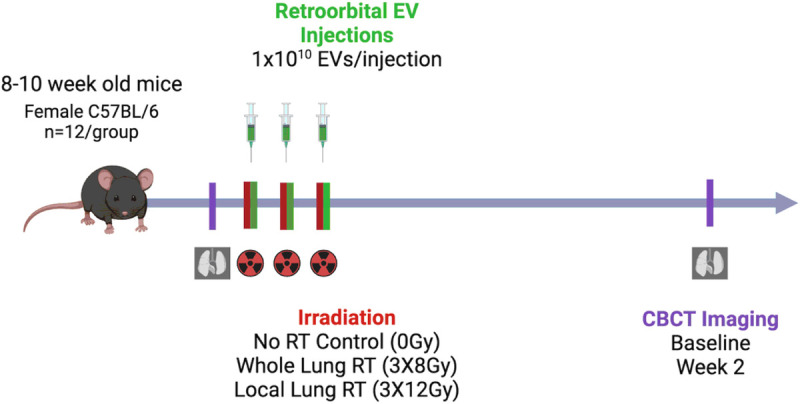
Schematic of Study Design.

With the aim of identifying radiation induced lung inflammation and lung injury, lung density was measured by CBCT at 2 weeks post-exposure, as shown in the study design figure (Fig. 1). Our previous results on animals irradiated with a single dose of 14.4 Gy showed a significant increase in lung density in the lung of irradiated animals 2 weeks post-exposure, measured after 2D manual contouring^7^. This increase in lung density was not observed in the animals treated with a single injection of hESC-derived EV. In the present study, at a similar time point and using the same 2D quantification method, no differences in HU were observed in any of the treatment groups (see Fig. 3), suggesting an absence of radiation-induced lung injury at early timepoint after the delivery of 3 × 8 Gy total lung or 3 × 12 Gy to the apex of the right-lung. All CBCT data was tested for normality (whole lung p = 0.0601, local irradiation: left lungs p = 0.007, right lungs p = 0.0776) and then either parametric or non-parametric ANOVA tests. In the whole lung irradiated cohort normality was shown (p = 0.0601) and ANOVA test showed no significant differences between groups (F(3,43) = 1.789, p = 0.1636). In the locally irradiated lungs the mean intensity was calculated for each lung separately and the distributions were not consistently Gaussian (left lung: p = 0.007, right lung: p = 0.0776). The Kruskal-Wallis test showed global significance (p = 0.0158, Kruskal-Wallis statistic = 17.26) but the multiple comparisons tests were all non-significant. No protective effect of hESC-derived EV could be confirmed with our experimental design. For this reason, and to rule out the possibility that the absence of difference in lung density could be due to the 2D quantification technique, we performed radiomics analysis on the same set of data.

### Principal Component Analysis indicates a distinction between irradiated groups and controls

A Principal Component Analysis (PCA) produced 70 principal components with the two most representative of the spread of the data comprising 32.6% (PC1) and 18.6% (PC2) of the variance respectively. The net change features from each animal were plotted against PC1 and PC2 as shown in Fig. 4. The figure indicates overlap of the control and unirradiated EV groups as well as overlap between the irradiated vehicle and the irradiated EV groups. However, a large proportion of the irradiated animals both with and without EV treatment, are distinct from the unirradiated groups. This suggests that a radiation effect is observed which is not mitigated by the injection of EVs. The plot suggests that a classifier could be trained to distinguish irradiated animals from controls with high accuracy.

### Machine learning models are capable of distinguishing irradiated, and EV treated animals from controls

Three supervised classification models were trained and tested on this dataset for a prediction task to determine if an animal had received a given treatment based on fewer than 20 radiomic features. Two feature selection algorithms - Feature Importance (FI) and Recursive Feature Elimination (RFE) – were compared and optimized for classification accuracy. RFE was the higher performing method and used to select the feature set for all 3 classifiers. The selected features are shown in Table 1. The radiation and EV classifiers were both binary classification models which utilized the full dataset but only trained on predicting one treatment type. The multi-class classification model (the treatment classifier) was trained to predict all 4 classes of treatment. As expected, the features selected for the treatment classifier overlapped with features selected for the two binary classifiers. However, no features were selected for all 3 models.

All the classification models were able to achieve good accuracy, statistically significantly greater than the no information rate. The highest performing classifier was the radiation classifier which achieved an accuracy of 94.29% and an area under the curve of 0.934. The ROC curves and metrics are shown in Fig. 5 and Table 2. The EV classifier achieved an accuracy of 85.71% and an AUC of 0.859. The treatment classifier attained an accuracy of 65.71% with all binary distinctions achieving an accuracy greater than the no information rate. The two classes that the treatment classifier was most accurate at distinguishing was between the control group and the radiation plus EV injection group. The worst performing distinction was between the radiated plus vehicle group and the radiation plus EV injection group. These results suggest therefore that a radiation effect is more evident than an EV effect alone and that no mitigation of the radiation effect from the EV treatment was observed.

### Feature Inspection reveals significant radiation effect in 3 features but no EV effect

All the radiomic features selected by either RFE or FI were inspected individually to identify group differences. Three features were found with significant differences between treatment groups using One-Way ANOVA and Bonferroni multiple comparisons tests. These features (as shown in Fig. 6) are GLDM Dependence Entropy from LLH wavelet image (F(3,66) = 8.019, p = 0.0001), Max 2D Diameter Row from the unfiltered image (F(3,66) = 3.959, p = 0.0117), and GLRLM Run Entropy also from the LLH wavelet image (F(3,66) = 4.639, p = 0.0053). Normality was confirmed in all three features (Dependence Entropy: p = 0.6798, Max 2D Diameter Row: p = 0.9792, Run Entropy: p = 0.7149). A difference between an unirradiated group and an irradiated group was observed in all 3 features but no difference between vehicle-injected and EV groups was observed, nor an EV sparing effect, in support of the indication by the PCA.

### Radiomic features identify changes in the locally irradiated lung

In the locally irradiated cohort, RFE was repeated, and the selected features inspected. The left and right lungs were split during image segmentation and a set of radiomic features were extracted for each lung individually. It was found that three radiomic features showed significant differences between groups as assessed by ANOVA (Fig. 7). These features were the intensity feature Kurtosis from the LLH wavelet image, the GLRLM Large Area High Gray Level Emphasis feature from the HLL wavelet image, and the Gray Level Non-Uniformity feature from the unfiltered image. The observed differences were only in the right lung and were predominantly between the unirradiated vehicle injected control lungs and the irradiated vehicle injected lungs. However, one feature (Large Area High Gray Level Emphasis) showed a surprising significant difference between the unirradiated vehicle group and the unirradiated EV treated group. In addition, no differences between the unirradiated vehicle injected control lungs and the irradiated EV treated lungs were seen, suggesting an EV sparing effect. Normality was verified for each feature and each lung individually (kurtosis: left p = 0.0663, right p = 0.1080; emphasis: left p = 0.5337, right = 0.5140; non-uniformity: left p = 0.0513, right p = 0.1593).

## Discussion

While deep learning shows increasing promise in classifying medical images and extracting features from huge datasets, the opacity of the decision-making being done by these algorithms hinders the translation into medical applications. It is especially the case for diagnosis and prognosis, where the logic at each step of the decision process is vital for the medical professional and patient to understand. Therefore, much attention is being drawn to the field of radiomics, where features are engineered and therefore can be replicated between institutions in efforts to both streamline workflow and derive a unified and comprehensible meaning. In addition, using traditional machine learning models such as decision tree classifiers or logistic regression to analyze these features instead of neural networks, allows for transparency throughout the image analysis process. These radiomic features are still being studied thoroughly to ensure they are robust, stable over time, and independent of the device used, image modality, imaging parameters, and other potential confounders. This study aimed to utilize radiomics in a manner that has not been investigated extensively to date, by using an animal model to determine if radiation toxicity could be distinguished using radiomic features.

The present results show no change in mean lung density measured in 2D slices from CBCT scans taken 2 weeks after exposure to hypofractionated doses of 3 × 8 Gy to the whole lungs and 3 × 12 Gy to the apex of the right lung, along with no impact from EV injection. However, from the 3D radiomics analysis, principal component analysis suggested a radiation effect but no EV effect which was supported by several radiomic features selected by Recursive Feature Elimination (RFE). These features were used to train and test a series of random forest machine learning classifiers which were able to predict both radiation and EV treatment with significant accuracy. The features selected by RFE for training and testing included predominantly texture and intensity features with a small number of spatial features. The data showed that the inter-group differences were not solely intensity changes or spatial changes and that a variety of features werefineeded to characterize a particular phenotype. A similar approach was conducted for a cohort of locally irradiated animals, except the left and right lungs were segmented individually before radiomic feature extraction. An inspection of the radiomic features indicated a radiation effect in the locally irradiated right lung that was not seen in the unirradiated left lung. Importantly, this approach uncovered a sparing effect of the EVs in the irradiated lung. The effect was found in features extracted from both the wavelet filtered images and the original image and was seen in both intensity and texture-based features. The data showed that the wavelet filtered images were able to amplify subtle patterns that may correspond with pathological endpoints.

The hurdle that is inevitably hit when analyzing large datasets is the risk of overfitting. By extracting > 800 features from a single image, it can be relatively easy to find features that fit any pattern the investigators choose. Therefore, care must be taken to minimize this risk as much as possible. This study was limited predominantly by the sample size and the lack of variety in the imaging machine and associated parameters. The risk of overfitting was minimized by randomly splitting the dataset into independent training and testing subsets of equal size. In addition, all preclinical data including animal IDs were removed from the dataset during model training and testing to prevent data leakage. In order to verify the correlation of these radiomic features with radiation-induced changes, further studies arefineeded using validated histologic endpoints (e.g. lung fibrosis, collagen deposition and alveolar thickness etc.) along with different imaging parameters and radiation doses, types, dose-rates, fractionation schedules etc.

In conclusion, this study is one of few to investigate if radiomic features are impacted by radiation-induced toxicity in the lungs of mice and shows the potential for radiomic features to support and identify subtle effects normally not captured through traditional metrics.

## Methods

### Animals

Animal experiments were approved by the Institutional Animal Care and Use Committee (IACUC) of the University of California Irvine, and performed and reported within institutional and ARRIVE guidelines. C57BL/6J mice were purchased from Charles River Laboratories and housed at the UCI vivarium from 10 weeks of age. The mice were kept in standard conditions with access to rodent chow and water *ad libitum*.

### Irradiation

The cohort (total of 72 mice, n = 12/group) consisted of 6 treatment groups. All animals were anesthetized with 2% isoflurane for irradiations and EV injections including the unirradiated controls. The irradiated animals either received whole thorax irradiation delivered in 3 fractions of 8 Gy, or local thoracic irradiation delivered to the apex of the right lung in 3 fractions of 12 Gy (see Fig. 1). In both cohorts, fractions were spaced out by 48 h. The right apex of the lung was chosen to minimize the dose delivered to the heart. Irradiation was delivered using a SmART + X-ray cabinet (Precision Inc.) at 225 kV, 13 mA, with a 0.3 mm copper filter and delivered with one (local apex exposure) or two (whole lung) opposite vertical beams after fluoroscan imaging to position the mice at the treatment isocenter. The prescribed doses were determined at 10 mm depth with a 15 mm circular (whole lung) or 5 mm circular (local apex exposure) collimated fields according to previous depth dose and dosimetric measurements in solid water phantoms with calibrated EBT3 Gafchromic films.

### Extracellular Vesicles

Human embryonic stem cell derived EV were derived from the hESC line H9 (WA09 Wicell Research Institute, Inc., Madison, WI) and extracted using biweekly ultracentrifugation and filtration as described previously^7^. Three retro-orbital injections of either 10^10^ hESC-derived EV or vehicle were performed immediately following each irradiation fraction or sham-irradiation while the mice were anesthetized. More details on the EV extraction and human stem cell culturing were already reported in^8^.

### Cone Beam Computed Tomography (CBCT) Analysis of Lung Fibrosis

Lung density was monitored for each animal using Cone-Beam Computed Tomography (CBCT) with the Precision SmART + small animal irradiator (80 kV; 1 mA), under isoflurane anesthesia. Images were acquired at baseline (the day of the first irradiation), 2 weeks following the last irradiation fraction. Lung contouring and reconstruction were performed using the Osirix Lite Software. Lung density was evaluated for each animal and each time point by Hounsfield Unit (HU) measurements. Values of ΔHU were calculated for each animal and time point by the formula: ΔHU_t(x)_ = HU_t(0)_ – HU_t(x)_.

### Radiomics Analysis

**Figure 2:**
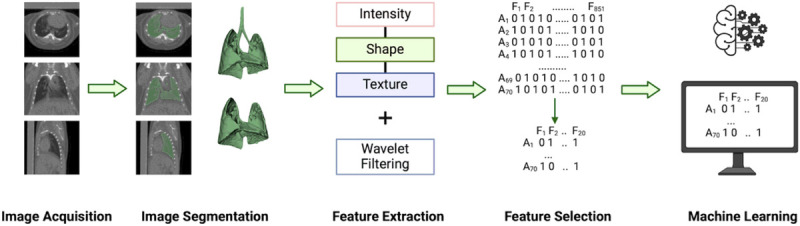
Radiomics analysis workflow for this study. Image segmentation and feature extraction were conducted in 3DSlicer. Feature selection and machine learning analysis were performed in R. No image preprocessing was conducted on this dataset prior to feature extraction.

**Figure 3:**
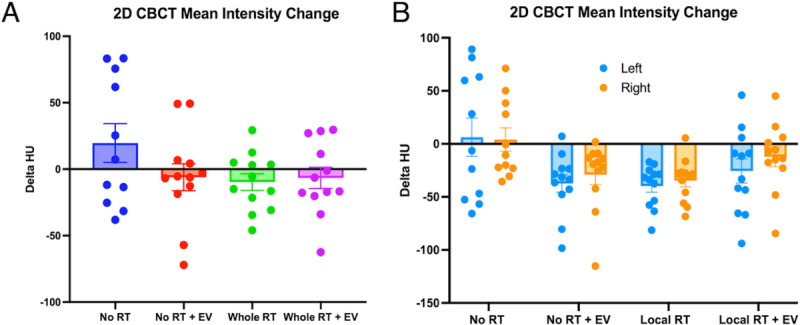
Change in Mean Intensity of CBCT lung images from a single 2D slice in Osirix Lite software. (A) The change in mean intensity measured for the whole lung 2 weeks after irradiation and/or EV injection. (B) The change in mean intensity measured separately for the left and right lungs in the locally irradiated cohort.

**Figure 4:**
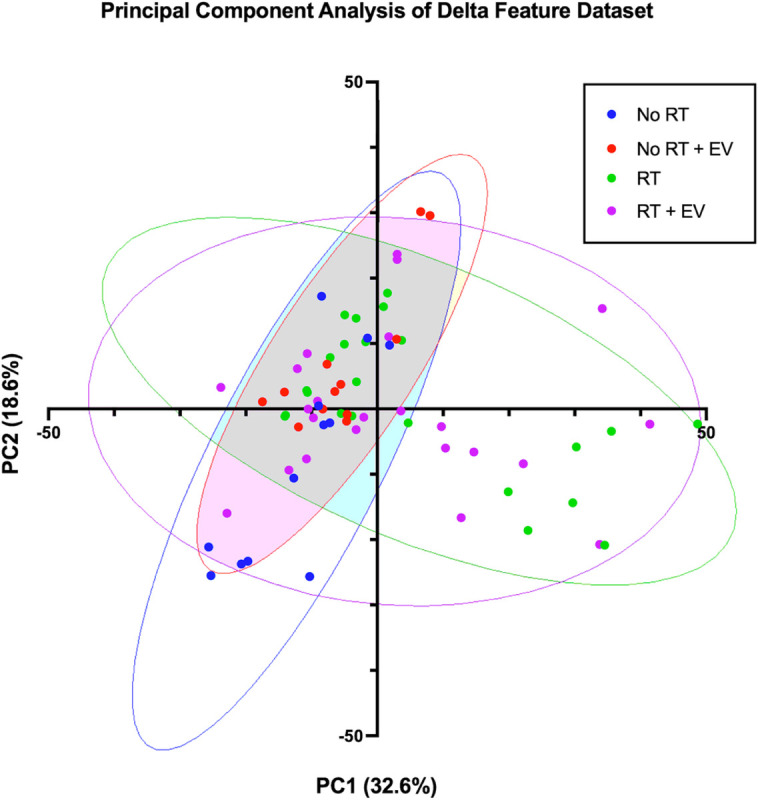
Scatter plot of the two principal components with the greatest contribution to the variance in the dataset. Each datapoints represents the net feature change of an animal from baseline to week 2. Treatment status was hidden during PCA and is shown by the ellipses (blue is the unirradiated and vehicle injected control group, red is the unirradiated and EV injected group, green is the irradiated and vehicle injected group and purple is the irradiated and EV injected group).

**Figure 5:**
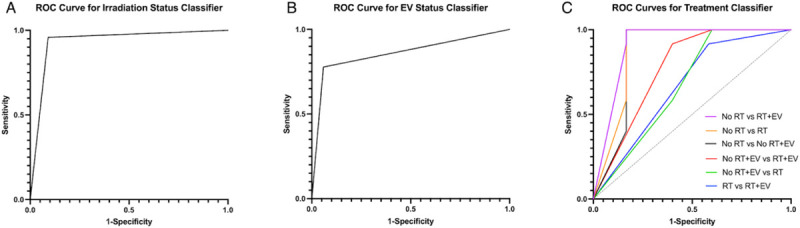
Receiver Operating Characteristic (ROC) Curves for the three classifiers. (A) Results of binary radiation classifier trained on features shown in first column of Table 1. Accuracy = 94.29%, area under the curve = 0.934. (B) Results of binary EV classifier trained on features shown in third column of Table 1. Accuracy = 85.71%, area under the curve = 0.859. (C) Results of multiclass classifier trained on features shown in second column of Table 1. Accuracy = 65.71%, mean area under the curve = 0.796.

**Figure 6:**
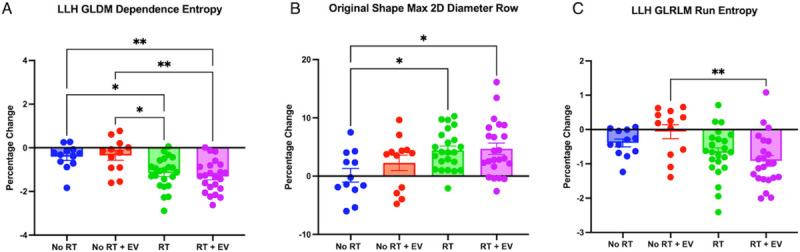
Box and whisker plots of the radiomic features which showed significant differences between treatment groups from one-way ANOVA and t-tests. (A) Plot of LLH filtered Gray Level Dependence Matrix Dependence Entropy (B) Plot of unfiltered image shape feature Maximum 2D Diameter Row (C) Plot of LLH filtered Gray Level Run Length Matrix Run Entropy.

**Figure 7:**
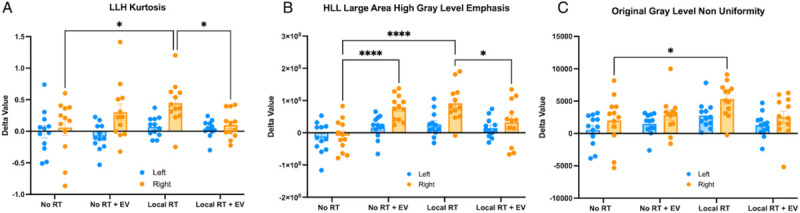
Bar graphs of a set of radiomic features which demonstrated a significant EV protective effect at 2 weeks in the locally irradiated animals. Blue indicates the unirradiated left lung and Orange indicates the irradiated right lung. (A) Plot of LLH Intensity feature Kurtosis (B) Plot of HLL filtered Gray Level Run Length Matrix feature Large Area High Gray Level Emphasis (C) Plot of unfiltered Gray Level Size Zone Matrix feature Gray Level Non Uniformity.

The stages of the radiomics analysis (see Fig. 2) were designed based on the traditional radiomics workflow as described in^9,10^. Image segmentation was performed semi-automatically as described below and image preprocessing was not performed before radiomic feature extraction. This was due to the relative homogenous nature of the imaging dataset being acquired on a single day, using a single machine and with one experimental cohort. The feature selection process included a Principal Component Analysis (PCA), Feature Importance, and Recursive Feature Elimination (RFE). The machine learning analysis included creation of random forest classifiers, trained and tested on independent subsets of the dataset.

### Image Segmentation

Whole lungs were segmented semi-automatically using the software 3DSlicer (version 4.11). A 3D mask was created with a range of −900 Hounsfield Units (HU) to −200 HU for all scans. This range includes functional lung tissue (−700 to −600 HU) and tissue with inflammation or fibrosis (−600 to −200 HU) while excluding air (−1000 HU) and surrounding soft tissue (0–300)^11,12^. Therefore, the threshold of −900 to −200 HU was selected in order to keep both healthy and inflamed lung parenchyma within the segmented volume while removing all surrounding soft tissue. The threshold range was optimized for 3DSlicer using a CT imaging dataset of healthy and fibrotic mouse lungs obtained at the CHUV in Lausanne, Switzerland and tested on a sample of images from the experimental cohort before settling on the final threshold values. After thresholding, the trachea, bronchi, pulmonary vessels and any artefacts of the segmentation were manually removed using the scissor tool in 3DSlicer. In order to divide the left and right lungs for the local irradiation classification model, the lungs were manually cut in 3D Slicer also using the scissor tool after segmentation of the whole lungs as described above.

### Feature Extraction

Radiomic features were calculated from the 3D mask using the 3D Slicer pyradiomics extension (version 3.0.1, python version 3.6). All the feature classes were extracted (Grey Length Dependence Matrix (GLDM), Shape based (2D and 3D), Grey Level Co-occurrence Matrix (GLCM), First Order Statistics, Grey Level Run Length Matrix (GLRLM), Grey Level Size Zone Matrix (GLSZM), and Neighbouring Grey Tone Difference Matrix (NGTDM)). The wavelet filter class was also applied to all scans to yield 8 derived images using the PyWavelets package (version 1.1.1). This method calculated 851 features for each scan which were combined into a single data frame and analyzed in R Studio (version 4.1.2).

### Feature Selection

The feature set contained data from image scans of the cohort taken at baseline and two weeks after all treatment. Net change features were calculated for each animal by subtracting f(0), the value at baseline, from f(1), the value at two weeks (in a similar way to the calculation of delta HU features described above). Therefore, the feature set contained a row of 851 features for each animal in the cohort (n = 70). The feature set was reduced first through removal of low variance features and removal of highly correlated features (greater than 95% correlation). This cleaned dataset contained 268 features and was used for the Principal Component Analysis (PCA) and feature selection methods described below.

Three methods of feature selection were performed. Firstly, PCA which allowed data visualization and dimensionality reduction. Secondly, feature importance was measured using the treatment status as the predictor, then the highest ranking 20 features were selected. The third method of feature selection was Recursive Feature Elimination (RFE) with a subset size of 20 features and cross-validation (n = 10). These values were selected after fine tuning of both the RFE and supervised classifiers. RFE was also repeated for each predictor variable and the results were used as the feature set for the supervised machine learning models. The model used for RFE was random forest, as with the supervised classifiers. The feature importance and RFE functions were installed from the caret package (version 6.0–93) and the PCA was conducted using the base R function (version 4.1.2).

### Machine Learning Analysis

Three classification models were trained and tested from this dataset, two binary classifiers aiming to predict radiation group or EV treatment group, and a multi-class classifier aiming to predict both radiation and EV treatment. Given the small size of the dataset, a 50:50 split was selected to divide the training set and test set. This way the chance of overfitting can be minimized while maximizing accuracy for both the training and test sets. Several models were evaluated and compared but the random forest model was ultimately chosen as it was consistently the most accurate. This is supported in the literature on radiomics of lung CTs^13^. The random forest function within the caret package (ranger) was used for all classifiers except for the EV treatment classifier which obtained greater accuracy with the base R function (rf).

Fine tuning of the random forest model was conducted, and the parameters were selected in order to optimize the Area Under the Curve (AUC) output. These parameters were training/test split (50:50), number of cross-validation folds (n = 5), and tune length (5).

## Statistical Analysis

Statistical analyses were carried out using GraphPad Prism (version 9) software. CBCT data were first evaluated for normal distribution using the Shapiro-Wilk test and then analyzed using one-way ANOVA followed by Bonferroni multiple comparison test. If the data were found to not fit a Gaussian distribution then the non-parametric Kruskal-Wallis ANOVA was conducted followed by the Dunn’s multiple comparison test. Data in the text are presented as means ± SEM, and all analyses considered a value of P ≤ 0.05 to be statistically significant.

## Figures and Tables

**Table 1:** Selected features determined by recursive feature elimination and utilized for the training and test datasets for the supervised classifiers. The wavelet prefix is included in the feature names.

Radiation Classifier	Treatment Group Classifier	EV Status Classifier
original Minor Axis Length		HHH Small Area Low Gray Level Emphasis
HLH Run Entropy		HLL Idmn
LLH Dependence Entropy		HLH first order Kurtosis
LHH MCC		HHH first order Skewness
LLH Sum Entropy		HLH first order Minimum
HLL MCC		HHL Size Zone Non-Uniformity Normalized
original Elongation	HHL Zone Entropy	
LLH Imc2	HLL first order Maximum	
LLL Contrast	LLH Run Entropy	
original Maximum 2D Diameter Row	LHH first order Mean	LLH Imc1
LHL Gray Level Non-Uniformity	original Max2D Diameter Slice	LHH Small Dependence Low Gray Level Emphasis
LLH Gray Level Non-Uniformity Normalized		HHL Small Area Low Gray Level Emphasis
HLL Correlation		HHH Joint Entropy
HLL Small Area High Gray Level Emphasis		HHH Imc2
LLH first order Skewness		
LLL Small Dependence Emphasis		

**Table 2:** Metrics of the 3 random forest classification models, each trained on the same 35 animals with the features shown in Table 1. The metrics are calculated from the test subset containing data from the remaining 35 animals.

	Radiation Status	EV Status	Treatment
**Accuracy (%)**	94.29	85.71	65.71
**AUC/Mean AUC**	0.934	0.859	0.796
**Sensitivity (%) /Mean Sensitivity**	90.91	94.12	64.17
**Specificity (%) /Mean Specificity**	95.83	77.78	86.96
**No information rate (%) (p-value)**	68.57 (2.6×10^−4^)	51.43 (2.3×10^−5^)	34.29 (1.5×10^−4^)

## Data Availability

The data that support the findings of this study are available from the corresponding author upon reasonable request.
